# A Reversed Trend: Care for Limited English Proficiency Patients in the Pediatric Emergency Department

**DOI:** 10.1155/2019/4832045

**Published:** 2019-03-11

**Authors:** David Greenky, Alyssa Levine, Scott E. Gillespie, Brittany Murray

**Affiliations:** ^1^Department of Pediatrics, Emory University School of Medicine, USA; ^2^Emory University School of Medicine, Atlanta, GA, USA; ^3^Division of Pediatric Emergency Medicine, Children's Healthcare of Atlanta/Emory University School of Medicine, USA

## Abstract

**Objectives:**

Previous studies in pediatric emergency departments (EDs) showed patients with limited English proficiency (LEP) had gaps in care compared with English-speaking patients. In 2010, the Joint Commission released patient-centered communication standards addressing these gaps. We evaluate the current care of LEP patients in the Children's Healthcare of Atlanta (CHOA) EDs.

**Methods:**

This was a retrospective cohort study of patients <18 years that presented to our EDs in 2016. Length of stay (LOS), change in triage status, return-visit rates, and hospital disposition were compared between patients who requested an interpreter and those who did not.

**Results:**

The population included 152,945 patients from 232,787 ED encounters in 2016. Interpreters were requested for 12.1% of encounters. For ED LOS, a model-adjusted difference of 0.77% was found between interpreter groups. For change in triage status, adjusted odds were 7% higher in the interpreter requested cohort. For ED readmission within 7 days, adjusted odds were 3% higher in the interpreter requested cohort. These effect sizes are small (ES < 0.2).

**Conclusions:**

Our study showed low ES of the differences in ED metrics between LEP and English-speaking patients, suggesting little clinical difference between the two groups. The impact of this improvement should be further studied.

## 1. Introduction

Limited English proficiency (LEP) is defined by the U.S. Census Bureau as the inability of an individual to speak English “very well.” [1] The LEP population has expanded rapidly in the United States over the past few decades, growing by 80% between 1990 and 2010 [2]. Even more dramatic increases were seen in certain states; the LEP population in Georgia increased by 379% during the same period [2].

This demographic change has been particularly evident in the healthcare system. In 2016, 12% of patients seen in Children's Healthcare of Atlanta (CHOA) Emergency Departments (EDs) indicated a preferred language other than English, compared to approximately 2.0% in 2002 [3]. It is important to note that, in our system, a child is considered LEP when their caregivers are LEP, even if the child is proficient in English. Previous studies have shown well-documented gaps in health outcomes for LEP patients who seek care in pediatric EDs. In the past, LEP patients have experienced extended length of stay (LOS), increased unplanned return rates, and increased rates of hospital admission [3–5]. Similar trends have been shown in adult/general EDs and internationally [5–7]. While the source of these disparities is likely multifactorial, issues surrounding communication are consistently recognized as a major contributing factor and have been the topic of investigation in many studies addressing the care of LEP patients [3, 4]. Poor communication and care disparities can lead to adverse events, low patient satisfaction, and excess cost [3, 4].

In 2010, the Joint Commission published communication guidelines titled “Advancing Effective Communication, Cultural Competence, and Patient- and Family-Centered Care” to improve overall communication with patients and, among other goals, ensure compliance with Title VI of the Civil Rights Act of 1964 [8]. This act prohibits discrimination based on race, color, or national origin for any program or activity that receives federal funding. This applies to any hospital that receives money from Medicaid, Medicare, or the Children's Health Insurance Program. If LEP patients cannot equally benefit from these programs, hospitals may be in violation of this act [8].

The Joint Commission guidelines aimed to avoid discrimination against LEP patients and close the gaps in care these patients experience. The guidelines emphasize linguistic and cultural understanding; moreover, they stress the importance of using qualified medical interpreters [9]. These guidelines were to be implemented by 2012, and a report was prepared by the Department of Health and Human Services to specifically guide hospitals to improve patient safety for LEP patients based on these guidelines [9].

The EDs within the CHOA system implemented the guidelines outlined by the Joint Commission in 2010 and continue to follow them. This is especially important given the increasingly global population in Metro Atlanta. A study conducted in the CHOA Egleston Hospital's ED in 2004 demonstrated that LEP patients were more likely to be triaged as “high acuity” and that LEP patients triaged as “moderate acuity” experienced increased admission rates compared to patients with English as a primary language [3]. Since the implementation of the Joint Commission guidelines, there has been no large-scale investigation to assess the success of the guidelines and the impact on care delivered to LEP patients in pediatric emergency medicine. This study seeks to determine the current differences in health outcomes between LEP patients and patients with English as a primary language in the CHOA EDs. Furthermore, it seeks to distinguish outcomes between Spanish-speaking patients who utilize interpreter services and LEP patients who speak less well-represented languages.

## 2. Materials and Methods

### 2.1. Study Protocol

This was a retrospective cohort study that looked at all patients aged 0-18 years that arrived in the three CHOA EDs (Hughes Spalding Hospital, Egleston Hospital, and Scottish Rite Hospital) between January 1, 2016, and December 31, 2016. These are pediatric hospitals located in Atlanta, GA, and seeing collectively more than 230,000 patients annually in their emergency departments. Patients were categorized as LEP if there was an interpreter requested during their encounter. The interpreter may have been requested by the patient, the patient's family, or the healthcare provider. The caregiver/guardian must agree for an interpreter to be used in the encounter. Patients who were dead on arrival or died in the ED and patients with no language or interpreter status listed in their charts were excluded. Beyond the exclusionary criteria, study variables of interest included the demographics race, ethnicity, gender, and age, as well as the hospital characteristics, insurance type, means of arrival, and maximum acuity.

### 2.2. Key Outcome Measures

Study outcomes were change in triage acuity, ED length of stay (LOS), readmission to the ED within seven days, and hospital disposition. Change in triage acuity was defined as a difference between initial and maximum acuity, recorded during the ED visit. Hospital disposition was described as a four-level characteristic (ICU, floor, transfer, and discharge) but principally analyzed as two-level variable (admission or transfer versus discharge). All study data were obtained from the CHOA electronic medical record (EPIC). IRB approval was received from both CHOA and Emory University.

### 2.3. Data Analysis

Statistical analyses were performed using SAS v9.4 (Cary, NC), and significance was evaluated two-sided at the 0.05 level. Demographic, clinical, and hospital characteristics were summarized using means and standard deviations, medians and interquartile ranges (IQR), or frequencies and percentages as appropriate. Baseline demographic summaries were calculated at the patient-level (N=152,945 records), whereas clinical and hospital values were computed at the encounter-level (N=232,787 records). Descriptive statistics were presented overall, by those requesting interpreter services versus not, and by those requesting a Spanish interpreter versus non-Spanish interpreter (a subset of the interpreter requested study group). Two-sample t-tests and Chi-square tests of independence were employed to evaluate bivariate associations between interpreter categories and study characteristics. When data were nonnormal, nonparametric equivalents were used (i.e., Kruskal-Wallis tests). Given the study's large sample and resulting likelihood for noninformative differences reaching statistical significance, Cohen's *d* effect sizes (ES) were further calculated as a measure of significance using the* tableone* package in CRAN R v3.3 (Vienna, Austria) [10]. Effect sizes were interpreted as small (ES of 0.2), moderate (ES of 0.5), and large (ES of 0.8) and principally interpreted, over p values, as indicators of clinical importance [10]. Finally, crude and adjusted associations between interpreter categories and the study outcomes were modeled using linear regression for ED LOS (after normality transformation) and logistic regression for change in acuity, ED readmission within 7 days, and hospital disposition. Linear- and logistic-adjusted associations controlled for age at baseline, insurance status, means of arrival, and maximum acuity as confounders and are presented as reverse-transformed least squares means and odds ratios with 95% confidence intervals, respectively. Regression-based values were interpreted as percent differences using Cohen's *d* thresholds for effect sizes.

## 3. Results

Table 1 describes the characteristics of 152,945 patients representing 232,787 ED encounters between January 1, 2016, and December 31, 2016, in the CHOA hospital system. Of these patients, 27,575 (18.0%) were self-identified as Hispanic or Latino ethnicity. 80,478 (52.6%) patients were African American and 53,706 (35.1%) patients were white and the median age was 5.4 years (IQR: 1.9-10.4).

Interpreter services were requested for at least one ED encounter in 18,572 (12.1%) patients. Of these patients, 15,582 (83.9%) were Latino or Hispanic, and 14,763 (79.5%) listed Spanish as their preferred language. Within the full study population, 17,341 (11.3%) patients indicated Spanish as their preferred language, and 133,677 (87.4%) cited English. Beyond Spanish and English, the next five preferred languages were Burmese 206 (0.1%), Amharic 152 (0.1%), Vietnamese 134 (0.09%), Portuguese 127 (0.08%), and Arabic 122 (0.08%).

Table 2 shows adjusted results for the outcome variables: (1) change in triage status, (2) ED LOS, (3) readmission within 7 days, and (4) hospital admission or transfer for patients that requested an interpreter compared to patients who did not request an interpreter. For ED LOS, a model-adjusted difference of 0.77% (1.2 minutes, 2.59 hours versus 2.61) was found between interpreter groups. For change in triage status, adjusted odds were 7% higher in the interpreter requested cohort (adjusted odds ratio (aOR) 1.07, 95% CI: 0.85, 1.35). For ED readmission within 7 days, adjusted odds were 3.0% higher in the interpreter requested cohort (aOR: 1.03, 95% CI: 0.98, 1.09). These ES are considered small and not clinically significant (ES < 0.2). Adjusted odds of hospital admission or transfer to another facility in interpreter requested patients were 6.0% higher than the adjusted odds in no interpreter requested patients (aOR: 1.06, 95% CI: 1.01, 1.11). This difference also corresponded to a small effect (ES < 0.2).

Table 3 considers admission and transfer prevalence, by patient acuity, as assigned by triage nurses in the interpreter requested versus no interpreter requested cohorts. High acuity patients (scores of 1-2) were compared with low and moderate acuity patients (scores of 3-5). Odds of hospital admittance/transfer were 9.53 times higher in high acuity patients relative to moderate/low acuity patients in the no interpreter requested cohort; likewise, odds of hospital admittance/transfer were 9.29 times higher in high acuity patients versus moderate/low in the interpreter requested group. The difference in odds ratio (OR) was 2.6% between the no interpreter and interpreter requested groups (9.53 versus 9.29), corresponding to no clinical difference in ORs for admission/transfer between the two groups.

Table 4 shows adjusted results for Spanish interpreter requested patients versus non-Spanish interpreter requested patients and is analogous to Table 2 For ED LOS, ED readmission within 7 days, and hospital disposition; there was no clinical significance between study groups (ES<0.2); however, adjusted odds of change in triage for Spanish interpreter requested patients were 43.0% higher than the adjusted odds for non-Spanish interpreter requested patients. This difference corresponded to a moderate effect size (ES<0.5), but change in triage rates in each group was very low, 0.3% and 0.2% for Spanish interpreters and non-Spanish interpreters, respectively.

## 4. Discussion

When considering the model-adjusted ED metrics of change in acuity, LOS, readmission, and ED disposition, our study showed small ES between LEP and English-speaking groups, suggesting there is little difference between the two populations with respect to these metrics. This is a reversed trend compared to previous studies in pediatric EM. In comparison to a 2004 study completed at CHOA's Egleston Hospital, our study showed that there was no clinically significant difference in admission/transfer rate between the two groups [3].

While there are likely multiple factors accounting for this change, we propose the successful implementation of the 2010 Joint Commission guidelines to be a driving force. These guidelines have inspired CHOA to grow its medical interpreter program. Interpreter usage in the pediatric ED has been shown to improve patient understanding and outcomes. A recent study showed interpreter usage during discharge improved the completeness of discharge instructions given and provider assessment of caregiver comprehension which the authors postulated led to better outcomes [11].

The three CHOA EDs have robust interpreter services available that have grown greatly in the past 15 years. This includes a dedicated in-person medical Spanish interpreting service, with increased availability during peak hours. For other languages, in-person interpreters from contracted companies are used whenever possible. In-person interpreters are familiar with the patients' communities and hospital procedures. This offers continuity that may further enhance communication between provider and patient, as the interpreters have a better understanding of the patients' needs and experiences within the hospital system. When an in-person interpreter is not available, a video or phone interpretation service is readily accessible. The interpreter department at CHOA has grown greatly since 2003. In 2003 there were 4 Spanish interpreters at CHOA and there are currently 37 interpreters. This growth coincides with the improvement that our study showed compared to the 2004 study regarding admission rates for LEP patients.

Low interpreter usage has been identified as a problem in the care of LEP patients. A 2016 study suggested some physicians are reluctant to utilize interpreters [12] and a more recent paper in 2018 suggested professional interpreters continue to be underutilized [13]. Providers at CHOA are encouraged to employ medical interpreters and document their use in the EMR and medical note. This is important, as in 2016 12.1% of patients requested an interpreter and deemed LEP compared to 2.0% in 2002. Interpreters can be requested by the patient or provider during multiple points in an encounter: registration, interaction with nurses, and interaction with physicians. On an institutional level, this both creates a culture that encourages a lower threshold for interpreter use and relieves some of the administrative burden associated with interpreter utilization. In our study 83.3% of patients that had a primary language other than English were documented as having requested an interpreter. Other studies have documented 10-50% interpreter usage for LEP patients [7]. These statistics should be compared with some caution as studies use various wording to document interpreter usage. No similar statistic was listed in the 2004 study at Egleston Children's Hospital.

Interestingly, our data suggested that non-LEP patients spent slightly more time in the ED than LEP patients. This difference was not clinically significant due to the low ES value and amounted to only a 1.2-minute difference.

The majority of LEP patients both in the United States and in this study are Spanish-speaking [9]. Previous studies showed that Spanish-speaking patients have increased Spanish resources compared to non-Spanish LEP patients and better health outcomes in the ED [9]. For this reason, the same ED metrics were compared between Spanish-speaking patients and other non-English-speaking patients. ED LOS and ED readmission within 7 days had a low ES, suggesting no clinical difference between the two groups. Spanish speakers had a lower admission rate compared to non-Spanish LEP patients, with an ES that suggested a small to moderate difference. A possible explanation is that lower-resourced non-Spanish-speaking patients are be admitted more frequently if providers are unsure of their ability to access resources in the community or return to care if needed.

Our study has some limitations. This is a retrospective study and data was collected using electronic medical records. 83.3% of patients listed as having a primary language other than English were documented as having requested an interpreter. It is unclear if the remaining 16.7% of patients did not use an interpreter or if one was used but not documented. Likewise, the EMR field reads, “interpreter requested”; however, there is no field to confirm that an interpreter was offered or used. While it is standard practice to offer every LEP patient an interpreter, this is not documented in the EMR. It is also unclear who requested the interpreter: the patient, family, or healthcare provider. There is likely a cohort of patients who declined an interpreter but would have been better served by using an interpreter. At times, although discouraged by hospital policies, family members or even clinicians may serve as interpreters. It is also unclear who was LEP in these encounters: patients, caregivers, or both. These subtleties are impossible to track in our current EMR but are an important consideration for future studies. Finally, this study was performed in a highly resourced academic setting and different results may be found in other centers.

## 5. Conclusion

In contrast to many previous studies performed in pediatric EDs, our study showed that differences in ED metrics between LEP and English-speaking patients have a low ES, suggesting little clinical difference between the two groups. While reasons for this change are likely multifactorial, improved communication with LEP patients as mandated by the 2010 Joint Commission communication guidelines and enforced by hospital culture, policies, and resources likely plays an important role. The potential impact of these changes, including the impact on adverse events, quality of care, patient satisfaction, and resource utilization, should be further studied.

## Figures and Tables

**Table 1:** Patient and encounter characteristics overall and by interpreter requested.

Characteristic	All Patients	Interpreter Request	No Interpreter Request	P-Value (ES^1)^
*Language-Related Patient Characteristics, N=152,945*				
Race				
White	53,706 (35.1%)	8,728 (47%)	44,978 (33.5%)	<0.001 (1.581)
African American	80,478 (52.6%)	1,107 (6%)	79,371 (59.1%)	
Asian	4,734 (3.1%)	803 (4.3%)	3,931 (2.9%)	
American Indian or Alaskan Native	637 (0.4%)	192 (1%)	445 (0.3%)	
Native Hawaiian or Pacific Islander	451 (0.3%)	187 (1%)	264 (0.2%)	
Other	16 (0.01%)	6 (0.03%)	10 (0.01%)	
Declined/Unknown	12,923 (8.5%)	7,549 (40.7%)	5,374 (4%)	
Ethnicity				
Not Hispanic or Latino	124,816 (81.6%)	2,930 (15.8%)	121,886 (90.7%)	<0.001 (2.286)
Hispanic or Latino	27,575 (18%)	15,582 (83.9%)	11,993 (8.9%)	
Decline/Unknown	554 (0.4%)	60 (0.3%)	494 (0.4%)	
Preferred Language				
English	133,677 (87.4%)	2,520 (13.6%)	131,157 (97.6%)	<0.001 (3.171)
Spanish	17,341 (11.3%)	14,763 (79.5%)	2,578 (1.9%)	
Other^2^	1,927 (1.3%)	1,289 (6.9%)	638 (0.5%)	

*Non-Language-Related Patient Characteristics, N=152,945*				
Sex – Male	80,682 (52.7%)	9,905 (53.3%)	70,777 (52.7%)	0.091 (0.013)
Age at Baseline				
Mean ± SD	6.5 ± 5.2	6.4 ± 4.9	6.5 ± 5.2	<0.001 (0.025)
Median (IQR), (Min, Max)	5.4 (1.9 - 10.4), (0, 18)	5.6 (2 - 10.1), (0, 18)	5.4 (1.9 - 10.5), (0, 18)	
Insurance Status				
Public	98,249 (64.2%)	16.011 (86.2%)	82,238 (61.2%)	<0.001 (0.772)
Private	42,754 (27.9%)	718 (3.9%)	42,036 (31.3%)	
Self-Pay/None	11,942 (7.8%)	1.843 (9.9%)	10,099 (7.5%)	

*Encounter Characteristics, N=232,787*				
Means of Arrival				
Air	523 (0.2%)	34 (0.1%)	489 (0.2%)	<0.001 (0.173)
Ambulance	24,552 (10.6%)	1,796 (6.4%)	22,756 (11.1%)	
Self/Caregiver	207,224 (89%)	26,388 (93.3%)	180,836 (88.4%)	
Other	454 (0.2%)	59 (0.2%)	395 (0.2%)	
Unknown	34 (0.01%)	2 (0.01%)	32 (0.02%)	
Maximum Acuity				
High (1-2)	42,201 (18.1%)	3,818 (13.5%)	38,383 (18.8%)	<0.001 (0.232)
Moderate (3)	79,301 (34.1%)	8,141 (28.8%)	71,160 (34.8%)	
Low (4-5)	111,109 (47.8%)	16,298 (57.7%)	94,811 (46.4%)	

*Outcomes*				
Change in Acuity – Yes^3^	693 (0.3%)	87 (0.3%)	606 (0.3%)	0.743 (0.002)
ED LOS (hours)				
Mean ± SD	2.8 ± 2.3	2.6 ± 1.8	2.9 ± 2.3	<0.001 (0.044)
Median (IQR), (Min, Max)	2.4 (1.6 - 3.6), (0.1, 182)	2.2 (1.5 - 3.3), (0.1, 61)	2.4 (1.6 - 3.6), (0.1, 182)	
ED Readmission within 7 Days – Yes	16,340 (7%)	1,999 (7.1%)	14,341 (7%)	0.728 (0.002)
Hospital Disposition				
Pediatric ICU	2,212 (0.9%)	221 (0.8%)	1,991 (1%)	
Floor Admission	25,809 (11.1%)	2,573 (9.1%)	23,236 (11.4%)	<0.001 (0.107)
Transfer facility	2,680 (1.2%)	161 (0.6%)	2,519 (1.2%)	
Discharge	202,086 (86.8%)	25,324 (89.6%)	176,762 (86.4%)	

^1^ES (standardized mean difference): <0.2, small, <0.5, moderate, <0.8, large.

^2^Top 5 other specified languages: Burmese (N=206, 0.13%), Amharic (N=152, 0.1%), Vietnamese (N=134, 0.09%), Portuguese (N=127, 0.08%), and Arabic (N=122, 0.08%).

^3^Measured by change from initial to max acuity during patient encounter.

**Table 2:** Adjusted outcome variables by interpreter needed.

Study Group	Crude Estimate (95% CI)	P-Value	Adjusted Estimate (95% CI)^1,3^	P-Value
*Change in Acuity*				
Interpreter Request	1.04 (0.83, 1.30)	0.740	1.07 (0.85, 1.35)	0.545
No Interpreter Request	Reference		Reference	

*ED LOS (hours)* ^*2*^				
Interpreter Request	2.19 (2.18, 2.21)	<0.001	2.59 (2.49, 2.69)	0.046
No Interpreter Request	2.37 (2.37, 2.38)		2.61 (2.51, 2.70)	

*ED Readmission within 7 Days*				
Interpreter Request	1.01 (0.96, 1.06)	0.727	1.03 (0.98, 1.09)	0.182
No Interpreter Request	Reference		Reference	

*ICU/Hospital Admission/Transfer*				
Interpreter Request	0.74 (0.71, 0.77)	<0.001	1.06 (1.01, 1.11)	0.014
No Interpreter Request	Reference		Reference	

^*∗*^ED LOS modeled using linear regression; ED readmission and ICU/hospital admission/transfer modeled using logistic regression.

^1^Adjusted Estimates account for age at baseline, insurance status, means of arrival, and maximum acuity.

^2^ED LOS (hours) was log-transformed prior to linear regression analysis and reverse-exponentiated for interpretation.

^3^0.77% difference in adjusted ED LOS hours between interpreter groups; 7%, 3%, and 6% differences in adjusted odds for (1) change in acuity, (2) ED readmission, and (3) ICU/hospital admittance/transfer, respectively.

**Table 3:** Admission rates by acuity and interpreter status.

Characteristic	N	High (1-2)	N	Moderate/Low (3-5)	OR (95% CI)^1^	P-Value
*Interpreter Request*						
ICU/Hospital Admission/Transfer	3,818	1,447 (37.9%)	24,439	1,506 (6.2%)	9.29 (8.55, 10.10)	<0.001
Discharged		2,371 (62.1%)		22,933 (93.8%)		

*No Interpreter Request*						
ICU/Hospital Admission/Transfer	38,383	16,070 (41.9%)	165,971	11,664 (7%)	9.53 (9.27, 9.80)	<0.001
Discharged		22,313 (58.1%)		154,307 (93%)		

^1^Breslow-Day-Tarone (BDT) test compares OR between interpreter requested and no interpreter requested groups. This p value was insignificant (OR 9.3 versus 9.5, p=0.579), indicating no difference in the odds of ICU/hospital admission/transfer for those with high acuity between interpreter groups.

^2^Mantel-Haenszel statistics showed higher odds for ICU/hospital admission/transfer in the high acuity patient group, after *adjustment for interpreter request status* (OR: 9.51; 95% CI: 9.26, 9.76; p<0.001).

**Table 4:** Outcome variables by interpreter needed.

Study Group	Crude Estimate (95% CI)	P-Value	Adjusted Estimate (95% CI)^1,3^	P-Value
*Change in Acuity*				
Spanish Interpreter	1.34 (0.75, 2.37)	0.323	1.43 (0.79, 2.61)	0.239
Non-Spanish Interpreter	Reference		Reference	

*ED LOS (hours)* ^*2*^				
Spanish Interpreter	2.17 (2.15, 2.19)	<0.001	2.42 (2.08, 2.81)	0.606
Non-Spanish Interpreter	2.29 (2.25, 2.32)		2.43 (2.09, 2.82)	

*ED Readmission within 7 Days*				
Spanish Interpreter	1.03 (0.92, 1.15)	0.640	1.08 (0.96, 1.21)	0.224
Non-Spanish Interpreter	Reference		Reference	

*ICU/Hospital Admission/Transfer*				
Spanish Interpreter	0.89 (0.81, 0.98)	0.014	1.13 (1.12, 1.13)	<0.001
Non-Spanish Interpreter	Reference		Reference	

^*∗*^ED LOS modeled using linear regression; ED readmission and hospital admission/transfer modeled using logistic regression.

^1^Adjusted estimates account for age at baseline, insurance status, means of arrival, and maximum acuity.

^2^ED LOS (hours) was log-transformed prior to linear regression analysis and reverse-exponentiated for interpretation.

^3^0.41% difference in adjusted ED LOS hours between interpreter groups; 43%, 8%, and 13% differences in adjusted odds for (1) change in acuity, (2) ED readmission, and (3) ICU/hospital admittance/transfer, respectively.

## Data Availability

Data was taken from EPIC EMR at Children's Healthcare of Atlanta. Dataset is available.
